# Crystal structure of the one-dimensional metal–organic polymer *catena*-poly[[tris(μ-2,4,6-tri­methyl­benzoato-κ^2^ *O*:*O*′)dizinc]-μ-2,4,6-tri­methyl­benzoato-κ^2^ *O*:*O*′]

**DOI:** 10.1107/S2056989014027418

**Published:** 2015-01-01

**Authors:** Masaki Yamamura, Tatsuya Nabeshima

**Affiliations:** a Graduate School of Pure and Applied Sciences, Tsukuba Research Center for Interdisciplinary Materials Science (TIMS), University of Tsukuba, 1-1-1, Tennodai, Tsukuba, Ibaraki 305-8571, Japan

**Keywords:** crystal structure, zinc cluster, metal–organic polymer, carboxyl­ate

## Abstract

The title complex, [Zn_2_(C_10_H_11_O_2_)_4_]_*n*_, has a one-dimensional polymeric structure. The asymmetric unit consists of two zinc atoms bridged by three 2,4,6-tri­methyl­benzoate anions and one bidentate bridging 2,4,6-trimethylbenzoate anion. The [Zn_2_(C_9_H_11_CO_2_)_3_] cluster units are inter­molecularly linked to form a one-dimensional polymer, which propagates in the direction of the crystallographic *b* axis. The Zn atoms adopt a tetra­hedral geometry. The Zn—O bond lengths in the intra­molecular bridges are slightly shorter than those in the inter­molecular bridges.

## Related literature   

For related polymeric complexes based on zinc benzoate, see: Clark & Kao (1948 ▸); Guseinov *et al.* (1984 ▸); Bijini *et al.* (2012 ▸); on zinc 2-chloro­benzoate, see: Clegg *et al.* (1990 ▸); and on zinc 2,3,5,6-tetra­methyl-1,4-benzene­dicarboxyl­ate, see: Braun *et al.* (2001 ▸).
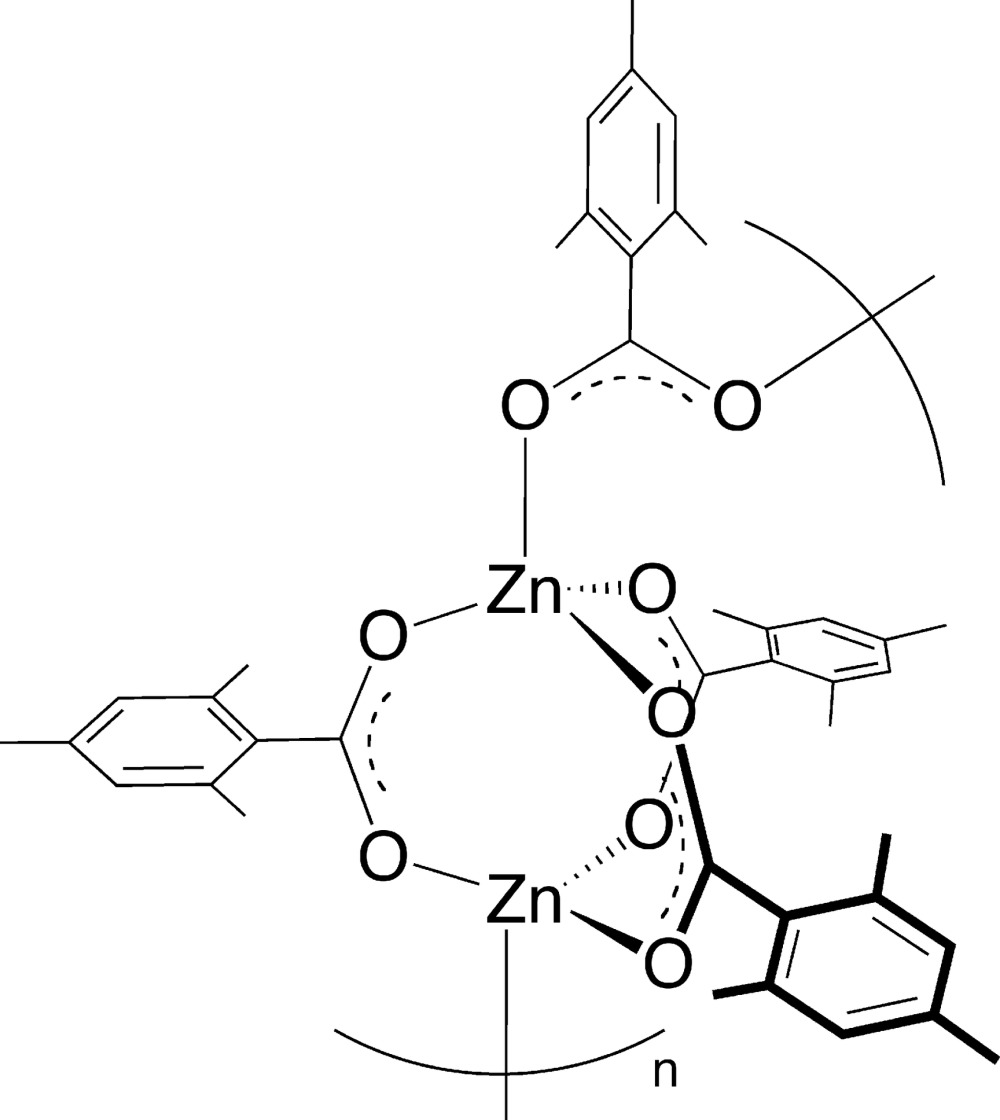


## Experimental   

### Crystal data  

[Zn_2_(C_10_H_11_O_2_)_4_]
*M* *_r_* = 783.49
Monoclinic, 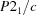

*a* = 12.0578 (12) Å
*b* = 14.5824 (15) Å
*c* = 22.275 (2) Å
β = 102.360 (1)°
*V* = 3825.9 (7) Å^3^
*Z* = 4
Mo *K*α radiation
μ = 1.30 mm^−1^
*T* = 120 K
0.20 × 0.10 × 0.05 mm

### Data collection  

Bruker APEXII CCD diffractometer
Absorption correction: multi-scan (*SADABS*; Bruker, 2007 ▸) *T*_min_ = 0.855, *T*_max_ = 0.937
21404 measured reflections
8867 independent reflections
7157 reflections with *I* > 2σ(*I*)
*R*_int_ = 0.026

### Refinement  

*R*[*F* ^2^ > 2σ(*F* ^2^)] = 0.034
*wR*(*F* ^2^) = 0.080
*S* = 1.01
8867 reflections
463 parameters
H-atom parameters constrained
Δρ_max_ = 0.78 e Å^−3^
Δρ_min_ = −0.39 e Å^−3^

### 

Data collection: *APEX2* (Bruker, 2007 ▸); cell refinement: *SAINT* (Bruker, 2007 ▸); data reduction: *SAINT*; program(s) used to solve structure: *SHELXS97* (Sheldrick, 2008 ▸); program(s) used to refine structure: *SHELXL2014* (Sheldrick, 2008 ▸); molecular graphics: *ORTEP-3 for Windows* (Farrugia, 2012 ▸); software used to prepare material for publication: *publCIF* (Westrip, 2010 ▸).

## Supplementary Material

Crystal structure: contains datablock(s) I, New_Global_Publ_Block. DOI: 10.1107/S2056989014027418/nk2228sup1.cif

Structure factors: contains datablock(s) I. DOI: 10.1107/S2056989014027418/nk2228Isup2.hkl

**Fig. 1.**
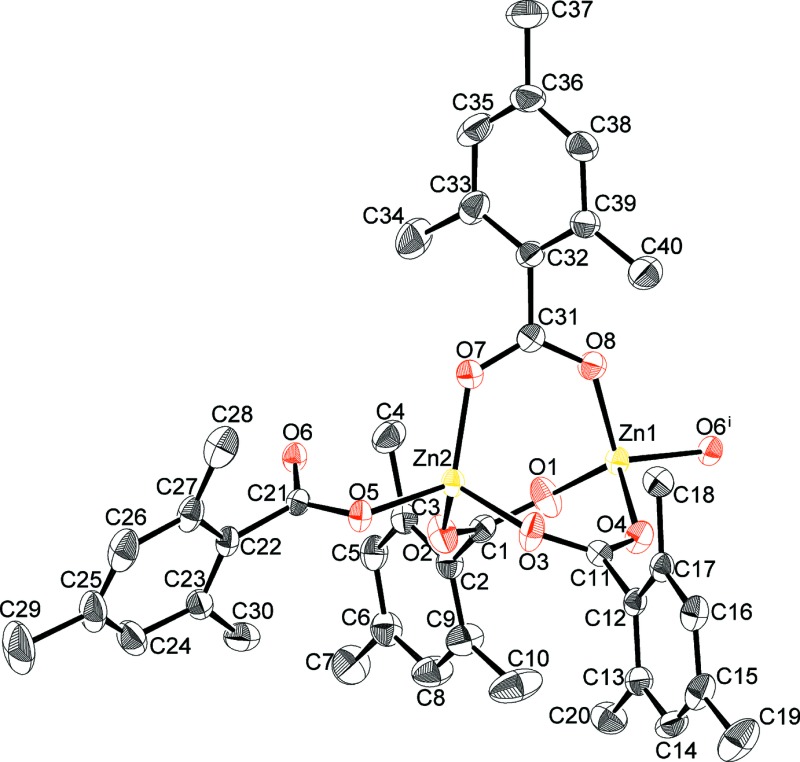
The molecular structure of the title complex. Displacement ellipsoids are drawn at the 50% probability level. Symmetry codes: (i) -x, y - 1/2, -z + 1/2.

**Fig. 2.**
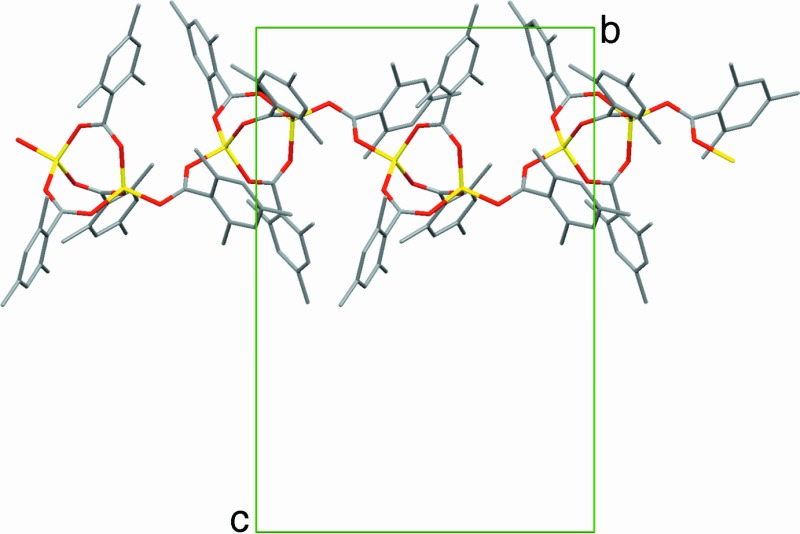
The one-dimensional polymeric chain in the crystal structure of the title compound in a view along the a axis.

Click here for additional data file.x y z . DOI: 10.1107/S2056989014027418/nk2228fig1.tif
The mol­ecular structure of the title complex. Displacement ellipsoids are drawn at the 50% probability level. Symmetry codes: (i) −*x*, *y* − 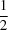, −*z* + 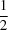.

a . DOI: 10.1107/S2056989014027418/nk2228fig2.tif
The one-dimensional polymeric chain in the crystal structure of the title compound in a view along the *a* axis.

CCDC reference: 1039507

Additional supporting information:  crystallographic information; 3D view; checkCIF report

## Figures and Tables

**Table 1:** Selected bond lengths ()

Zn1O1	1.9361(16)
Zn1O8	1.9382(15)
Zn1O4	1.9436(15)
Zn1O6^i^	1.9532(15)
Zn2O7	1.9258(16)
Zn2O2	1.9363(16)
Zn2O3	1.9492(16)
Zn2O5	1.9532(15)

Symmetry code: (i) 
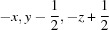
.

## supplementary crystallographic information

### S1. Comment

Coordination polymers composed of zinc carboxyl­ates have emerged as tremendously inter­esting crystalline materials because of their application in catalysis, gas storage, and sensing. Although there are a large number of reports of coordination polymers composed of zinc carboxyl­ates, there are only 21 examples having zinc dinuclear part bridged by three carboxyl­ate ligands in CCDC database. The title complex, zinc 2,4,6-tri­methyl­benzoate, (Fig. 1), crystallizes in a monoclinic unit cell with a single dinuclear-zinc-complex pair in the asymmetric unit, which are bridged by three *µ*-2,4,6-tri­methyl­benzoate anions. The zinc dinuclear parts are inter­molecularly linked by the other 2,4,6-tri­methyl­benzoate anion to form one-dimensional polymer (Fig. 2) which propagates in the direction of the crystallographic *b* axis. Zinc benzoate (Clark *et al.* (1948); Guseinov *et al.* (1984); Bijini *et al.* (2012)) and zinc 2-chloro­benzoate (Clegg *et al.* (1990)) exhibited the same polymeric structure. The Zn—O bond lengths in intra­molecular bridges, Zn1—O1, Zn1—O4, Zn1—O8, Zn2—O2, Zn2—O3, and Zn2—O7 are slightly shorter than those in inter­molecular bridges, Zn1—O6^i^ and Zn2—O5.

### S2. Synthesis and crystallization

To an ether solution (20 mL) of 2,4,6-tri­methyl­benzoic acid (1.0 g, 6.1 mmol) was added an Et_2_Zn/hexane solution (1.0 M, 3.0 mL, 3.0 mmol) slowly. The white precipitate was washed with ether, and then reprecipitation from chloro­form/methanol gave a colorless powder of zinc 2,4,6-tri­methyl­benzoate (1.1 g, 93%). Single crystals were obtained by slow evaporation of methanol/chloro­form solution of the title complex. Anal. Calcd for C_40_H_44_O_8_Zn_2_: C, 61.31; H, 5.66. Found: C, 61.14; H, 5.66. ^1^H NMR (400 MHz, DMSO-d_6_) 2.20 (s, 3H), 2.22 (s, 6H), 6.77 (s, 2H). ^13^C (100 MHz, DMSO-d_6_) 19.64 (CH_3_), 20.67 (CH_3_), 127.43 (CH), 132.81 (C), 135.56 (C), 137.06 (C), 175.76 (C).

### S3. Refinement

All of the positional parameters and thermal parameters of non-hydrogen atoms were anisotropically refined on F^2^ by the full-matrix least-squares method. Hydrogen atoms were placed at the calculated positions ((C—H = 0.95 Å (phenyl) or 0.98 Å (methyl)) and refined as riding on their corresponding carbon atoms with Uiso(H) = 1.5 times Ueq(C) for methyl H atoms and = 1.2 times Ueq(C) for other H atoms.

### Crystal data

**Table:**

[Zn_2_(C_10_H_11_O_2_)_4_]	*F*(000) = 1632
*M**_r_* = 783.49	*D*_x_ = 1.360 Mg m^−^^3^
Monoclinic, *P*2_1_/*c*	Mo *K*α radiation, λ = 0.71073 Å
*a* = 12.0578 (12) Å	Cell parameters from 1351 reflections
*b* = 14.5824 (15) Å	θ = 2.7–27.5°
*c* = 22.275 (2) Å	µ = 1.30 mm^−^^1^
β = 102.360 (1)°	*T* = 120 K
*V* = 3825.9 (7) Å^3^	Block, pale yellow
*Z* = 4	0.20 × 0.10 × 0.05 mm

### Data collection

**Table:**

Bruker APEXII CCD diffractometer	8867 independent reflections
Radiation source: fine-focus sealed tube	7157 reflections with *I* > 2σ(*I*)
Graphite monochromator	*R*_int_ = 0.026
φ and ω scans	θ_max_ = 28.5°, θ_min_ = 1.7°
Absorption correction: multi-scan (*SADABS*; Bruker, 2007)	*h* = −15→15
*T*_min_ = 0.855, *T*_max_ = 0.937	*k* = −19→16
21404 measured reflections	*l* = −29→28

### Refinement

**Table:**

Refinement on *F*^2^	0 restraints
Least-squares matrix: full	Hydrogen site location: inferred from neighbouring sites
*R*[*F*^2^ > 2σ(*F*^2^)] = 0.034	H-atom parameters constrained
*wR*(*F*^2^) = 0.080	*w* = 1/[σ^2^(*F*_o_^2^) + (0.020*P*)^2^ + 5.*P*] where *P* = (*F*_o_^2^ + 2*F*_c_^2^)/3
*S* = 1.01	(Δ/σ)_max_ < 0.001
8867 reflections	Δρ_max_ = 0.78 e Å^−^^3^
463 parameters	Δρ_min_ = −0.39 e Å^−^^3^

### Special details

**Table:**

Geometry. All e.s.d.'s (except the e.s.d. in the dihedral angle between two l.s. planes) are estimated using the full covariance matrix. The cell e.s.d.'s are taken into account individually in the estimation of e.s.d.'s in distances, angles and torsion angles; correlations between e.s.d.'s in cell parameters are only used when they are defined by crystal symmetry. An approximate (isotropic) treatment of cell e.s.d.'s is used for estimating e.s.d.'s involving l.s. planes.

### Fractional atomic coordinates and isotropic or equivalent isotropic displacement parameters (Å^2^)

**Table:**

	*x*	*y*	*z*	*U*_iso_*/*U*_eq_	
Zn1	−0.01963 (2)	−0.09027 (2)	0.23624 (2)	0.02038 (7)	
Zn2	0.03822 (2)	0.10799 (2)	0.18232 (2)	0.02097 (7)	
O1	0.06415 (15)	−0.02033 (12)	0.30507 (7)	0.0356 (4)	
O2	0.13211 (15)	0.10089 (12)	0.26440 (8)	0.0350 (4)	
O3	0.07929 (15)	0.01158 (11)	0.13049 (8)	0.0333 (4)	
O4	0.07284 (14)	−0.12268 (11)	0.17787 (7)	0.0282 (4)	
O5	0.09977 (13)	0.21646 (10)	0.14961 (7)	0.0229 (3)	
O6	0.04628 (12)	0.29611 (10)	0.22260 (7)	0.0228 (3)	
O7	−0.12449 (13)	0.10862 (11)	0.17198 (8)	0.0286 (4)	
O8	−0.16060 (13)	−0.03770 (11)	0.19109 (7)	0.0276 (3)	
C1	0.12488 (19)	0.04947 (16)	0.30840 (10)	0.0242 (5)	
C2	0.19581 (19)	0.07447 (16)	0.36990 (10)	0.0256 (5)	
C3	0.15013 (19)	0.13166 (16)	0.40868 (10)	0.0267 (5)	
C4	0.0311 (2)	0.16725 (19)	0.38925 (12)	0.0380 (6)	
H4A	0.0053	0.1902	0.4253	0.057*	
H4B	−0.0191	0.1176	0.3702	0.057*	
H4C	0.0293	0.2172	0.3596	0.057*	
C5	0.2184 (2)	0.15481 (17)	0.46520 (11)	0.0305 (5)	
H5	0.1881	0.1928	0.4924	0.037*	
C6	0.3292 (2)	0.12416 (18)	0.48299 (11)	0.0332 (5)	
C7	0.4023 (2)	0.1525 (2)	0.54398 (12)	0.0455 (7)	
H7A	0.4707	0.1142	0.5530	0.068*	
H7B	0.3596	0.1446	0.5764	0.068*	
H7C	0.4241	0.2170	0.5421	0.068*	
C8	0.3712 (2)	0.0667 (2)	0.44354 (12)	0.0409 (6)	
H8	0.4467	0.0444	0.4556	0.049*	
C9	0.3057 (2)	0.0407 (2)	0.38663 (11)	0.0368 (6)	
C10	0.3514 (3)	−0.0230 (3)	0.34406 (14)	0.0597 (10)	
H10A	0.3071	−0.0798	0.3387	0.090*	
H10B	0.4312	−0.0371	0.3618	0.090*	
H10C	0.3456	0.0068	0.3041	0.090*	
C11	0.09609 (17)	−0.07343 (15)	0.13581 (9)	0.0208 (4)	
C12	0.14756 (18)	−0.11860 (14)	0.08808 (10)	0.0214 (4)	
C13	0.26351 (19)	−0.13914 (16)	0.10146 (11)	0.0270 (5)	
C14	0.3086 (2)	−0.18030 (17)	0.05554 (12)	0.0340 (6)	
H14	0.3873	−0.1945	0.0638	0.041*	
C15	0.2426 (2)	−0.20120 (17)	−0.00179 (12)	0.0343 (6)	
C16	0.1284 (2)	−0.17891 (16)	−0.01349 (11)	0.0295 (5)	
H16	0.0825	−0.1924	−0.0527	0.035*	
C17	0.07876 (19)	−0.13739 (15)	0.03051 (10)	0.0232 (4)	
C18	−0.04636 (19)	−0.11609 (17)	0.01647 (11)	0.0293 (5)	
H18A	−0.0809	−0.1380	−0.0249	0.044*	
H18B	−0.0573	−0.0497	0.0186	0.044*	
H18C	−0.0822	−0.1467	0.0466	0.044*	
C19	0.2929 (3)	−0.2497 (2)	−0.04983 (14)	0.0524 (8)	
H19A	0.3047	−0.3146	−0.0389	0.079*	
H19B	0.3658	−0.2214	−0.0518	0.079*	
H19C	0.2408	−0.2445	−0.0900	0.079*	
C20	0.3372 (2)	−0.11762 (19)	0.16336 (12)	0.0372 (6)	
H20A	0.4102	−0.1493	0.1677	0.056*	
H20B	0.2993	−0.1382	0.1957	0.056*	
H20C	0.3502	−0.0513	0.1670	0.056*	
C21	0.10079 (17)	0.28918 (14)	0.18022 (9)	0.0193 (4)	
C22	0.17023 (19)	0.36796 (15)	0.16578 (10)	0.0228 (4)	
C23	0.2758 (2)	0.38501 (16)	0.20402 (11)	0.0283 (5)	
C24	0.3370 (2)	0.46119 (19)	0.19157 (13)	0.0400 (6)	
H24	0.4082	0.4744	0.2178	0.048*	
C25	0.2967 (3)	0.51814 (19)	0.14191 (15)	0.0460 (7)	
C26	0.1934 (3)	0.49777 (18)	0.10388 (13)	0.0434 (7)	
H26	0.1659	0.5359	0.0693	0.052*	
C27	0.1280 (2)	0.42316 (16)	0.11443 (11)	0.0311 (5)	
C28	0.0159 (2)	0.40370 (19)	0.07240 (11)	0.0394 (6)	
H28A	−0.0106	0.4586	0.0482	0.059*	
H28B	−0.0395	0.3865	0.0968	0.059*	
H28C	0.0245	0.3532	0.0447	0.059*	
C29	0.3633 (4)	0.6017 (2)	0.1296 (2)	0.0733 (12)	
H29A	0.4307	0.6087	0.1627	0.110*	
H29B	0.3155	0.6564	0.1276	0.110*	
H29C	0.3868	0.5939	0.0904	0.110*	
C30	0.3245 (2)	0.32251 (19)	0.25705 (12)	0.0350 (6)	
H30A	0.2625	0.2951	0.2731	0.053*	
H30B	0.3737	0.3579	0.2896	0.053*	
H30C	0.3689	0.2739	0.2429	0.053*	
C31	−0.19067 (18)	0.04227 (16)	0.17330 (10)	0.0235 (4)	
C32	−0.31529 (19)	0.06039 (16)	0.15257 (11)	0.0259 (5)	
C33	−0.3641 (2)	0.13265 (18)	0.17911 (12)	0.0345 (6)	
C34	−0.2952 (3)	0.1926 (2)	0.22803 (14)	0.0510 (8)	
H34A	−0.2465	0.1543	0.2590	0.077*	
H34B	−0.3463	0.2288	0.2475	0.077*	
H34C	−0.2479	0.2340	0.2095	0.077*	
C35	−0.4808 (2)	0.14777 (19)	0.15916 (13)	0.0421 (7)	
H35	−0.5151	0.1957	0.1777	0.051*	
C36	−0.5479 (2)	0.09607 (19)	0.11378 (14)	0.0404 (7)	
C37	−0.6736 (2)	0.1147 (2)	0.09255 (18)	0.0588 (9)	
H37A	−0.6870	0.1497	0.0541	0.088*	
H37B	−0.7005	0.1501	0.1240	0.088*	
H37C	−0.7148	0.0564	0.0857	0.088*	
C38	−0.4969 (2)	0.02634 (18)	0.08789 (13)	0.0378 (6)	
H38	−0.5418	−0.0094	0.0561	0.045*	
C39	−0.38183 (19)	0.00636 (17)	0.10665 (11)	0.0298 (5)	
C40	−0.3316 (2)	−0.07034 (19)	0.07582 (12)	0.0392 (6)	
H40A	−0.3901	−0.0960	0.0427	0.059*	
H40B	−0.3032	−0.1183	0.1060	0.059*	
H40C	−0.2689	−0.0466	0.0588	0.059*	

### Atomic displacement parameters (Å^2^)

**Table:**

	*U*^11^	*U*^22^	*U*^33^	*U*^12^	*U*^13^	*U*^23^
Zn1	0.02529 (13)	0.01865 (13)	0.01827 (12)	−0.00039 (10)	0.00701 (10)	0.00168 (9)
Zn2	0.02660 (13)	0.01742 (13)	0.01969 (12)	−0.00098 (10)	0.00674 (10)	0.00089 (9)
O1	0.0473 (11)	0.0369 (10)	0.0214 (8)	−0.0171 (8)	0.0048 (7)	−0.0021 (7)
O2	0.0414 (10)	0.0307 (10)	0.0271 (9)	−0.0078 (8)	−0.0054 (7)	0.0072 (7)
O3	0.0543 (11)	0.0195 (9)	0.0321 (9)	0.0012 (7)	0.0223 (8)	−0.0009 (7)
O4	0.0386 (9)	0.0244 (8)	0.0254 (8)	0.0040 (7)	0.0155 (7)	0.0034 (6)
O5	0.0344 (8)	0.0176 (8)	0.0196 (7)	−0.0015 (6)	0.0119 (6)	−0.0009 (6)
O6	0.0301 (8)	0.0190 (8)	0.0222 (7)	−0.0010 (6)	0.0117 (6)	−0.0020 (6)
O7	0.0262 (8)	0.0233 (9)	0.0357 (9)	−0.0012 (6)	0.0051 (7)	0.0013 (7)
O8	0.0258 (8)	0.0250 (9)	0.0318 (9)	0.0017 (6)	0.0056 (7)	0.0064 (7)
C1	0.0262 (11)	0.0235 (12)	0.0228 (11)	0.0036 (9)	0.0052 (9)	−0.0019 (9)
C2	0.0293 (12)	0.0247 (12)	0.0218 (11)	−0.0018 (9)	0.0030 (9)	−0.0020 (9)
C3	0.0294 (12)	0.0234 (12)	0.0265 (11)	−0.0015 (9)	0.0040 (9)	−0.0003 (9)
C4	0.0370 (14)	0.0353 (15)	0.0398 (15)	0.0079 (11)	0.0037 (11)	−0.0109 (11)
C5	0.0404 (14)	0.0255 (12)	0.0259 (12)	−0.0017 (10)	0.0078 (10)	−0.0043 (9)
C6	0.0367 (13)	0.0331 (14)	0.0276 (12)	−0.0026 (10)	0.0018 (10)	−0.0030 (10)
C7	0.0469 (16)	0.0494 (18)	0.0343 (14)	0.0001 (13)	−0.0044 (12)	−0.0113 (13)
C8	0.0309 (13)	0.0546 (18)	0.0334 (14)	0.0087 (12)	−0.0014 (11)	−0.0059 (13)
C9	0.0346 (14)	0.0457 (16)	0.0285 (13)	0.0063 (11)	0.0036 (10)	−0.0073 (11)
C10	0.0433 (17)	0.090 (3)	0.0418 (17)	0.0257 (17)	−0.0007 (13)	−0.0247 (17)
C11	0.0209 (10)	0.0231 (11)	0.0185 (10)	−0.0018 (8)	0.0040 (8)	0.0004 (8)
C12	0.0271 (11)	0.0164 (10)	0.0231 (10)	0.0006 (8)	0.0105 (9)	0.0017 (8)
C13	0.0273 (11)	0.0237 (12)	0.0309 (12)	0.0009 (9)	0.0084 (9)	0.0074 (10)
C14	0.0296 (12)	0.0308 (13)	0.0469 (15)	0.0094 (10)	0.0200 (11)	0.0111 (11)
C15	0.0497 (15)	0.0261 (13)	0.0344 (13)	0.0074 (11)	0.0249 (12)	0.0047 (10)
C16	0.0428 (14)	0.0248 (12)	0.0233 (11)	0.0012 (10)	0.0127 (10)	−0.0007 (9)
C17	0.0292 (11)	0.0188 (11)	0.0236 (11)	−0.0008 (9)	0.0099 (9)	0.0031 (9)
C18	0.0289 (12)	0.0314 (13)	0.0270 (12)	−0.0014 (10)	0.0047 (10)	0.0021 (10)
C19	0.074 (2)	0.0479 (18)	0.0473 (17)	0.0219 (16)	0.0398 (16)	0.0048 (14)
C20	0.0307 (13)	0.0427 (16)	0.0354 (14)	−0.0011 (11)	0.0011 (11)	0.0102 (12)
C21	0.0233 (10)	0.0176 (10)	0.0165 (10)	0.0018 (8)	0.0030 (8)	0.0021 (8)
C22	0.0323 (12)	0.0169 (10)	0.0231 (10)	0.0000 (9)	0.0147 (9)	−0.0005 (8)
C23	0.0311 (12)	0.0251 (12)	0.0328 (12)	−0.0030 (9)	0.0162 (10)	−0.0060 (10)
C24	0.0401 (15)	0.0350 (15)	0.0519 (17)	−0.0132 (12)	0.0252 (13)	−0.0122 (13)
C25	0.0620 (19)	0.0264 (14)	0.0623 (19)	−0.0104 (13)	0.0412 (16)	−0.0030 (13)
C26	0.068 (2)	0.0270 (14)	0.0449 (16)	0.0043 (13)	0.0328 (15)	0.0103 (12)
C27	0.0475 (15)	0.0213 (12)	0.0290 (12)	0.0037 (10)	0.0183 (11)	0.0037 (9)
C28	0.0595 (17)	0.0349 (15)	0.0248 (12)	0.0085 (12)	0.0111 (12)	0.0088 (11)
C29	0.098 (3)	0.0382 (19)	0.103 (3)	−0.0248 (18)	0.064 (2)	0.0000 (19)
C30	0.0263 (12)	0.0432 (16)	0.0347 (13)	0.0017 (11)	0.0044 (10)	−0.0050 (11)
C31	0.0275 (11)	0.0250 (12)	0.0186 (10)	0.0016 (9)	0.0067 (9)	−0.0011 (9)
C32	0.0250 (11)	0.0247 (12)	0.0307 (12)	0.0031 (9)	0.0119 (9)	0.0062 (9)
C33	0.0409 (14)	0.0282 (13)	0.0396 (14)	0.0054 (11)	0.0200 (11)	0.0050 (11)
C34	0.0606 (19)	0.0425 (17)	0.0541 (18)	0.0074 (14)	0.0214 (15)	−0.0155 (14)
C35	0.0457 (16)	0.0355 (15)	0.0542 (17)	0.0160 (12)	0.0306 (14)	0.0129 (13)
C36	0.0262 (12)	0.0380 (15)	0.0604 (18)	0.0035 (11)	0.0167 (12)	0.0193 (13)
C37	0.0294 (14)	0.052 (2)	0.099 (3)	0.0091 (13)	0.0207 (16)	0.0269 (19)
C38	0.0275 (13)	0.0332 (14)	0.0512 (16)	−0.0012 (11)	0.0050 (11)	0.0125 (12)
C39	0.0252 (11)	0.0280 (13)	0.0363 (13)	0.0009 (9)	0.0069 (10)	0.0069 (10)
C40	0.0344 (14)	0.0392 (15)	0.0392 (15)	0.0040 (11)	−0.0024 (11)	−0.0083 (12)

### Geometric parameters (Å, º)

**Table:**

Zn1—O1	1.9361 (16)	C18—H18B	0.9800
Zn1—O8	1.9382 (15)	C18—H18C	0.9800
Zn1—O4	1.9436 (15)	C19—H19A	0.9800
Zn1—O6^i^	1.9532 (15)	C19—H19B	0.9800
Zn2—O7	1.9258 (16)	C19—H19C	0.9800
Zn2—O2	1.9363 (16)	C20—H20A	0.9800
Zn2—O3	1.9492 (16)	C20—H20B	0.9800
Zn2—O5	1.9532 (15)	C20—H20C	0.9800
O1—C1	1.247 (3)	C21—C22	1.497 (3)
O2—C1	1.252 (3)	C22—C23	1.394 (3)
O3—C11	1.258 (3)	C22—C27	1.402 (3)
O4—C11	1.259 (3)	C23—C24	1.394 (3)
O5—C21	1.259 (2)	C23—C30	1.508 (3)
O6—C21	1.265 (2)	C24—C25	1.385 (4)
O6—Zn1^ii^	1.9532 (15)	C24—H24	0.9500
O7—C31	1.259 (3)	C25—C26	1.381 (4)
O8—C31	1.260 (3)	C25—C29	1.517 (4)
C1—C2	1.497 (3)	C26—C27	1.393 (4)
C2—C9	1.387 (3)	C26—H26	0.9500
C2—C3	1.396 (3)	C27—C28	1.497 (4)
C3—C5	1.391 (3)	C28—H28A	0.9800
C3—C4	1.500 (3)	C28—H28B	0.9800
C4—H4A	0.9800	C28—H28C	0.9800
C4—H4B	0.9800	C29—H29A	0.9800
C4—H4C	0.9800	C29—H29B	0.9800
C5—C6	1.384 (3)	C29—H29C	0.9800
C5—H5	0.9500	C30—H30A	0.9800
C6—C8	1.386 (4)	C30—H30B	0.9800
C6—C7	1.510 (3)	C30—H30C	0.9800
C7—H7A	0.9800	C31—C32	1.498 (3)
C7—H7B	0.9800	C32—C33	1.398 (3)
C7—H7C	0.9800	C32—C39	1.400 (3)
C8—C9	1.395 (4)	C33—C35	1.400 (4)
C8—H8	0.9500	C33—C34	1.501 (4)
C9—C10	1.513 (4)	C34—H34A	0.9800
C10—H10A	0.9800	C34—H34B	0.9800
C10—H10B	0.9800	C34—H34C	0.9800
C10—H10C	0.9800	C35—C36	1.377 (4)
C11—C12	1.494 (3)	C35—H35	0.9500
C12—C17	1.397 (3)	C36—C38	1.378 (4)
C12—C13	1.398 (3)	C36—C37	1.512 (4)
C13—C14	1.393 (3)	C37—H37A	0.9800
C13—C20	1.505 (3)	C37—H37B	0.9800
C14—C15	1.386 (4)	C37—H37C	0.9800
C14—H14	0.9500	C38—C39	1.391 (3)
C15—C16	1.384 (3)	C38—H38	0.9500
C15—C19	1.514 (3)	C39—C40	1.506 (3)
C16—C17	1.391 (3)	C40—H40A	0.9800
C16—H16	0.9500	C40—H40B	0.9800
C17—C18	1.506 (3)	C40—H40C	0.9800
C18—H18A	0.9800		
			
O1—Zn1—O8	116.97 (8)	H19A—C19—H19B	109.5
O1—Zn1—O4	112.40 (7)	C15—C19—H19C	109.5
O8—Zn1—O4	108.30 (7)	H19A—C19—H19C	109.5
O1—Zn1—O6^i^	100.69 (7)	H19B—C19—H19C	109.5
O8—Zn1—O6^i^	111.41 (6)	C13—C20—H20A	109.5
O4—Zn1—O6^i^	106.46 (6)	C13—C20—H20B	109.5
O7—Zn2—O2	119.20 (7)	H20A—C20—H20B	109.5
O7—Zn2—O3	108.31 (7)	C13—C20—H20C	109.5
O2—Zn2—O3	110.45 (8)	H20A—C20—H20C	109.5
O7—Zn2—O5	114.23 (7)	H20B—C20—H20C	109.5
O2—Zn2—O5	101.74 (7)	O5—C21—O6	121.68 (19)
O3—Zn2—O5	101.43 (6)	O5—C21—C22	118.11 (18)
C1—O1—Zn1	132.64 (15)	O6—C21—C22	120.21 (18)
C1—O2—Zn2	130.29 (15)	C23—C22—C27	121.4 (2)
C11—O3—Zn2	135.35 (15)	C23—C22—C21	119.0 (2)
C11—O4—Zn1	127.61 (15)	C27—C22—C21	119.6 (2)
C21—O5—Zn2	116.12 (13)	C24—C23—C22	118.3 (2)
C21—O6—Zn1^ii^	125.61 (14)	C24—C23—C30	120.3 (2)
C31—O7—Zn2	128.93 (15)	C22—C23—C30	121.5 (2)
C31—O8—Zn1	133.69 (15)	C25—C24—C23	121.8 (3)
O1—C1—O2	125.5 (2)	C25—C24—H24	119.1
O1—C1—C2	117.8 (2)	C23—C24—H24	119.1
O2—C1—C2	116.7 (2)	C26—C25—C24	118.5 (2)
C9—C2—C3	121.8 (2)	C26—C25—C29	120.3 (3)
C9—C2—C1	119.0 (2)	C24—C25—C29	121.2 (3)
C3—C2—C1	119.2 (2)	C25—C26—C27	122.3 (3)
C5—C3—C2	118.0 (2)	C25—C26—H26	118.9
C5—C3—C4	121.5 (2)	C27—C26—H26	118.9
C2—C3—C4	120.5 (2)	C26—C27—C22	117.8 (2)
C3—C4—H4A	109.5	C26—C27—C28	120.7 (2)
C3—C4—H4B	109.5	C22—C27—C28	121.5 (2)
H4A—C4—H4B	109.5	C27—C28—H28A	109.5
C3—C4—H4C	109.5	C27—C28—H28B	109.5
H4A—C4—H4C	109.5	H28A—C28—H28B	109.5
H4B—C4—H4C	109.5	C27—C28—H28C	109.5
C6—C5—C3	122.0 (2)	H28A—C28—H28C	109.5
C6—C5—H5	119.0	H28B—C28—H28C	109.5
C3—C5—H5	119.0	C25—C29—H29A	109.5
C5—C6—C8	118.4 (2)	C25—C29—H29B	109.5
C5—C6—C7	120.7 (2)	H29A—C29—H29B	109.5
C8—C6—C7	120.9 (2)	C25—C29—H29C	109.5
C6—C7—H7A	109.5	H29A—C29—H29C	109.5
C6—C7—H7B	109.5	H29B—C29—H29C	109.5
H7A—C7—H7B	109.5	C23—C30—H30A	109.5
C6—C7—H7C	109.5	C23—C30—H30B	109.5
H7A—C7—H7C	109.5	H30A—C30—H30B	109.5
H7B—C7—H7C	109.5	C23—C30—H30C	109.5
C6—C8—C9	121.8 (2)	H30A—C30—H30C	109.5
C6—C8—H8	119.1	H30B—C30—H30C	109.5
C9—C8—H8	119.1	O7—C31—O8	125.3 (2)
C2—C9—C8	118.1 (2)	O7—C31—C32	117.2 (2)
C2—C9—C10	120.3 (2)	O8—C31—C32	117.5 (2)
C8—C9—C10	121.6 (2)	C33—C32—C39	120.4 (2)
C9—C10—H10A	109.5	C33—C32—C31	119.2 (2)
C9—C10—H10B	109.5	C39—C32—C31	120.4 (2)
H10A—C10—H10B	109.5	C32—C33—C35	118.0 (3)
C9—C10—H10C	109.5	C32—C33—C34	121.9 (2)
H10A—C10—H10C	109.5	C35—C33—C34	120.1 (2)
H10B—C10—H10C	109.5	C33—C34—H34A	109.5
O3—C11—O4	125.1 (2)	C33—C34—H34B	109.5
O3—C11—C12	116.79 (18)	H34A—C34—H34B	109.5
O4—C11—C12	118.08 (19)	C33—C34—H34C	109.5
C17—C12—C13	121.6 (2)	H34A—C34—H34C	109.5
C17—C12—C11	118.95 (19)	H34B—C34—H34C	109.5
C13—C12—C11	119.4 (2)	C36—C35—C33	122.7 (2)
C14—C13—C12	117.7 (2)	C36—C35—H35	118.7
C14—C13—C20	121.1 (2)	C33—C35—H35	118.7
C12—C13—C20	121.2 (2)	C35—C36—C38	117.8 (2)
C15—C14—C13	122.3 (2)	C35—C36—C37	121.6 (3)
C15—C14—H14	118.9	C38—C36—C37	120.6 (3)
C13—C14—H14	118.9	C36—C37—H37A	109.5
C16—C15—C14	118.3 (2)	C36—C37—H37B	109.5
C16—C15—C19	120.6 (3)	H37A—C37—H37B	109.5
C14—C15—C19	121.1 (2)	C36—C37—H37C	109.5
C15—C16—C17	122.0 (2)	H37A—C37—H37C	109.5
C15—C16—H16	119.0	H37B—C37—H37C	109.5
C17—C16—H16	119.0	C36—C38—C39	122.4 (3)
C16—C17—C12	118.1 (2)	C36—C38—H38	118.8
C16—C17—C18	120.4 (2)	C39—C38—H38	118.8
C12—C17—C18	121.44 (19)	C38—C39—C32	118.7 (2)
C17—C18—H18A	109.5	C38—C39—C40	119.4 (2)
C17—C18—H18B	109.5	C32—C39—C40	121.9 (2)
H18A—C18—H18B	109.5	C39—C40—H40A	109.5
C17—C18—H18C	109.5	C39—C40—H40B	109.5
H18A—C18—H18C	109.5	H40A—C40—H40B	109.5
H18B—C18—H18C	109.5	C39—C40—H40C	109.5
C15—C19—H19A	109.5	H40A—C40—H40C	109.5
C15—C19—H19B	109.5	H40B—C40—H40C	109.5
			
Zn1—O1—C1—O2	11.9 (4)	Zn2—O5—C21—O6	−12.9 (3)
Zn1—O1—C1—C2	−167.94 (16)	Zn2—O5—C21—C22	166.21 (14)
Zn2—O2—C1—O1	12.8 (4)	Zn1^ii^—O6—C21—O5	−168.98 (14)
Zn2—O2—C1—C2	−167.40 (16)	Zn1^ii^—O6—C21—C22	11.9 (3)
O1—C1—C2—C9	91.1 (3)	O5—C21—C22—C23	−101.5 (2)
O2—C1—C2—C9	−88.7 (3)	O6—C21—C22—C23	77.6 (3)
O1—C1—C2—C3	−89.4 (3)	O5—C21—C22—C27	78.7 (3)
O2—C1—C2—C3	90.8 (3)	O6—C21—C22—C27	−102.2 (2)
C9—C2—C3—C5	0.3 (4)	C27—C22—C23—C24	2.8 (3)
C1—C2—C3—C5	−179.1 (2)	C21—C22—C23—C24	−177.0 (2)
C9—C2—C3—C4	179.9 (2)	C27—C22—C23—C30	−176.3 (2)
C1—C2—C3—C4	0.4 (3)	C21—C22—C23—C30	3.9 (3)
C2—C3—C5—C6	1.1 (4)	C22—C23—C24—C25	−1.5 (4)
C4—C3—C5—C6	−178.4 (2)	C30—C23—C24—C25	177.6 (2)
C3—C5—C6—C8	−1.8 (4)	C23—C24—C25—C26	−0.4 (4)
C3—C5—C6—C7	178.4 (2)	C23—C24—C25—C29	179.0 (3)
C5—C6—C8—C9	1.1 (4)	C24—C25—C26—C27	1.1 (4)
C7—C6—C8—C9	−179.1 (3)	C29—C25—C26—C27	−178.3 (3)
C3—C2—C9—C8	−1.0 (4)	C25—C26—C27—C22	0.1 (4)
C1—C2—C9—C8	178.5 (2)	C25—C26—C27—C28	179.5 (2)
C3—C2—C9—C10	178.5 (3)	C23—C22—C27—C26	−2.1 (3)
C1—C2—C9—C10	−2.0 (4)	C21—C22—C27—C26	177.7 (2)
C6—C8—C9—C2	0.3 (4)	C23—C22—C27—C28	178.5 (2)
C6—C8—C9—C10	−179.2 (3)	C21—C22—C27—C28	−1.7 (3)
Zn2—O3—C11—O4	11.1 (4)	Zn2—O7—C31—O8	11.4 (3)
Zn2—O3—C11—C12	−169.00 (16)	Zn2—O7—C31—C32	−169.20 (15)
Zn1—O4—C11—O3	12.8 (3)	Zn1—O8—C31—O7	13.8 (3)
Zn1—O4—C11—C12	−167.06 (14)	Zn1—O8—C31—C32	−165.51 (15)
O3—C11—C12—C17	−77.9 (3)	O7—C31—C32—C33	−53.1 (3)
O4—C11—C12—C17	102.0 (2)	O8—C31—C32—C33	126.3 (2)
O3—C11—C12—C13	100.6 (2)	O7—C31—C32—C39	126.1 (2)
O4—C11—C12—C13	−79.5 (3)	O8—C31—C32—C39	−54.5 (3)
C17—C12—C13—C14	−0.8 (3)	C39—C32—C33—C35	1.1 (3)
C11—C12—C13—C14	−179.3 (2)	C31—C32—C33—C35	−179.6 (2)
C17—C12—C13—C20	179.3 (2)	C39—C32—C33—C34	−179.4 (2)
C11—C12—C13—C20	0.8 (3)	C31—C32—C33—C34	−0.2 (4)
C12—C13—C14—C15	−0.2 (4)	C32—C33—C35—C36	−1.7 (4)
C20—C13—C14—C15	179.7 (2)	C34—C33—C35—C36	178.9 (3)
C13—C14—C15—C16	0.9 (4)	C33—C35—C36—C38	0.6 (4)
C13—C14—C15—C19	−177.3 (2)	C33—C35—C36—C37	−179.2 (3)
C14—C15—C16—C17	−0.6 (4)	C35—C36—C38—C39	0.9 (4)
C19—C15—C16—C17	177.6 (2)	C37—C36—C38—C39	−179.2 (2)
C15—C16—C17—C12	−0.3 (3)	C36—C38—C39—C32	−1.4 (4)
C15—C16—C17—C18	−178.7 (2)	C36—C38—C39—C40	−179.4 (2)
C13—C12—C17—C16	1.1 (3)	C33—C32—C39—C38	0.3 (3)
C11—C12—C17—C16	179.6 (2)	C31—C32—C39—C38	−178.9 (2)
C13—C12—C17—C18	179.4 (2)	C33—C32—C39—C40	178.3 (2)
C11—C12—C17—C18	−2.1 (3)	C31—C32—C39—C40	−0.9 (3)

Symmetry codes: (i) −*x*, *y*−1/2, −*z*+1/2; (ii) −*x*, *y*+1/2, −*z*+1/2.

## References

[bb1] Bijini, B. R., Prasanna, S., Deepa, M., Nair, C. M. K. & Babu, K. R. (2012). *Spectrochim. Acta Part A*, **97**, 1002–1006. 10.1016/j.saa.2012.07.107 22925975

[bb2] Braun, M. E., Steffek, C. D., Kim, J., Rasmussen, P. G. & Yaghi, O. M. (2001). *Chem. Commun.* pp. 2532–2533.

[bb3] Bruker (2007). *APEX2*, *SAINT* and *SADABS*. Bruker AXS Inc., Madison, Wisconsin, USA.

[bb4] Clark, G. L. & Kao, H. (1948). *J. Am. Chem. Soc.* **70**, 2151–2154.

[bb5] Clegg, W., Harbron, D. R., Hunt, P. A., Little, I. R. & Straughan, B. P. (1990). *Acta Cryst.* C**46**, 750–753.

[bb6] Farrugia, L. J. (2012). *J. Appl. Cryst.* **45**, 849–854.

[bb7] Guseinov, G. A., Musaev, F. N., Usubaliev, B. T., Amiraslanov, I. R. & Mamedov, Kh. S. (1984). *Koord. Khim.* **10**, 117–122.

[bb8] Sheldrick, G. M. (2008). *Acta Cryst.* A**64**, 112–122. 10.1107/S0108767307043930 18156677

[bb9] Westrip, S. P. (2010). *J. Appl. Cryst.* **43**, 920–925.

