# Supplementary figures and images for: Crystal structure of the one-dimensional metal–organic polymer catena-poly[[tris(μ-2,4,6-tri­methyl­benzoato-κ2 O:O′)dizinc]-μ-2,4,6-tri­methyl­benzoato-κ2 O:O′]
Source: Acta Crystallogr E Crystallogr Commun. 2015 Jan 1;71(Pt 1):m14–5. doi: 10.1107/S2056989014027418 (PMC4331921; doi:10.1107/S2056989014027418)

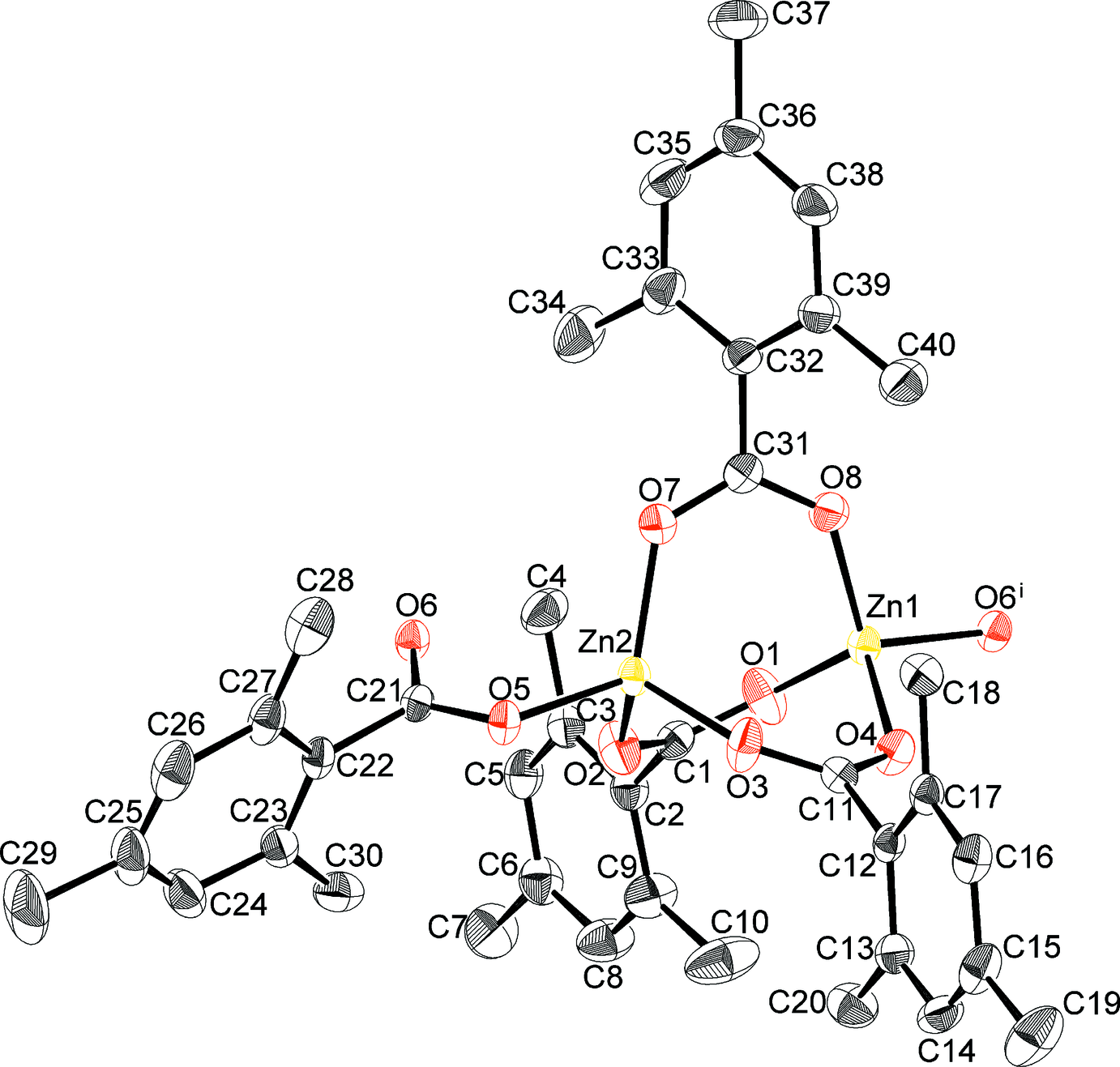

Supplement: Supplementary file 3 [file e-71-00m14-fig1.tif]

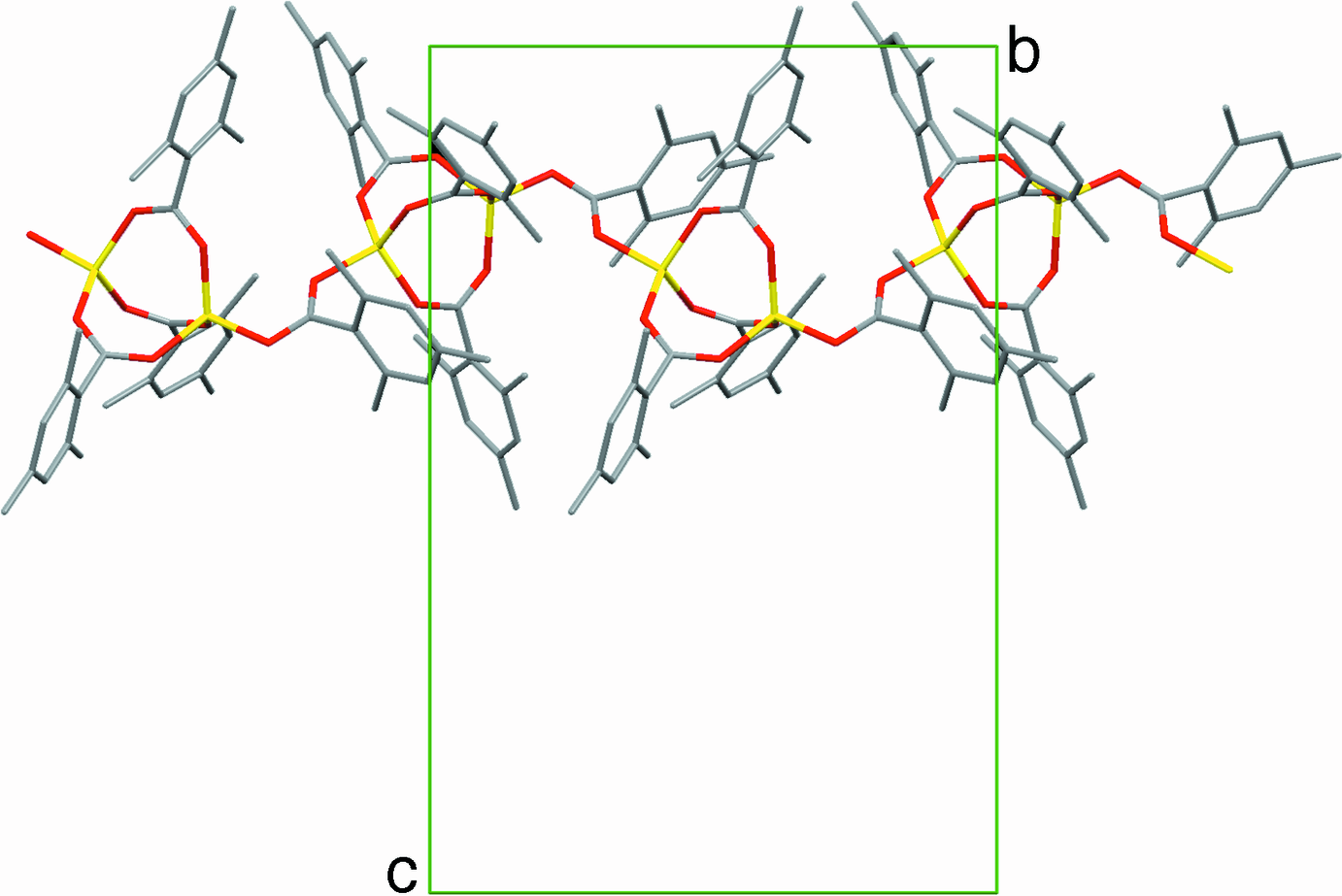

Supplement: Supplementary file 4 [file e-71-00m14-fig2.tif]
